# The Effect of Vaginal Er:YAG Laser Therapy on Pelvic Floor Symptoms in Women With Stress Urinary Incontinence: A Single‐Center Cohort Study

**DOI:** 10.1002/lsm.70001

**Published:** 2025-02-19

**Authors:** Massimiliano Lia, Pavel Pilát, Laura Weydandt, Kaven Baessler, Tom Kempe, Bahriye Aktas

**Affiliations:** ^1^ Department of Gynecology University Hospital of Leipzig Leipzig Germany; ^2^ Pelvic Floor Center Franziskus and St. Joseph Hospital Berlin Germany

**Keywords:** Er:YAG laser, mixed urinary incontinence, stress urinary incontinence, vaginal laser

## Abstract

**Objectives:**

To examine the effect of vaginal erbium:yttrium‐aluminum‐garnet (Er:YAG) laser therapy on pelvic floor symptoms in women with stress urinary incontinence (SUI).

**Methods:**

This was a prospective, single‐arm interventional study applying three Er:YAG laser treatments to patients with SUI. The German version of the validated Australian Pelvic Floor Questionnaire was used to quantify pelvic floor symptoms (bladder function, bowel function, sexual function, pelvic organ prolapse) over different time points (baseline, posttreatment, 3 months, and 6 months after treatment). Subgroups were compared to examine which factors influenced symptom development after the treatment.

**Results:**

A total of 50 women received three Er:YAG laser treatments and had their symptoms evaluated at all time points of the study. All symptom complex scores, especially bladder function and sexual function, showed statistically significant improvements lasting for the whole study period. However, score improvements of bowel function and prolapse symptoms were likely not clinically meaningful, as they were below the minimal important difference (MID) of this questionnaire. Moreover, sexual function scores improved less when women additionally had urgency symptoms (i.e., mixed urinary incontinence [MUI]; *p* = 0.036).

**Conclusion:**

Vaginal Er:YAG laser therapy has the potential to improve pelvic floor symptoms linked to bladder function and sexual function in women with SUI. However, the positive effect on sexual function was lower if the women had MUI.

## 1 Introduction

Female stress urinary incontinence (SUI) represents a relevant health problem with a prevalence of 4%−35% [1]. It is characterized by involuntary urine loss when pressure is applied on the bladder (i.e., physical exertion, coughing, laughing) and may be classified as mixed urinary incontinence (MUI) in the presence of additional urgency symptoms [2]. SUI may be a benign condition, but it influences several aspects of daily life and may even limit social activities [3]. The initial treatment for SUI encompasses lifestyle changes (i.e., weight reduction, fluid intake regulation) and pelvic floor muscle training [4]. However, while being noninvasive, these measures require guidance by professionals, depend on the patient's compliance, and may not be sufficient even if executed under perfect conditions [5, 6, 7]. Surgical procedures, such as colposuspension and vaginal sling procedures, have been shown to give long‐term results and high patient satisfaction [8]. However, these procedures may have serious adverse events in rare cases [9].

The non‐ablative erbium:yttrium‐aluminum‐garnet (Er:YAG) laser has been suggested to improve symptoms of SUI by delivering heat‐pulses into the vaginal tissue, thus inducing collagen remodeling, neocollagenesis, and neoelastogenesis [10, 11, 12]. In fact, previous studies observed that Er:YAG‐laser therapy led to an improvement of symptoms, a sonographically detectable decrease in bladder neck mobility and a change in vaginal topography in SUI‐patients [13, 14, 15, 16, 17, 18, 19].

Nevertheless, most of these studies are based on relatively small cohorts and the evidence about which factors influence treatment results is scarce. Additionally, previous studies mainly report and analyze cumulative questionnaire‐scores, which may give the impression of precision and objectivity due to its numeric nature. However, cumulative scores are only a rough approximation of the severity of the patients' complaints [20] and do not give specific information about specific symptoms. Consequently, reporting patient‐centered outcomes (i.e., outcomes meaningful to patients and caregivers [21]) after Er:YAG laser therapy would provide additional valuable information.

This study aims to examine the effect of vaginal Er:YAG laser therapy in patients with SUI by means of both cumulative questionnaire‐scores and specific questionnaire‐items. Additionally, this study explores if certain subgroups of SUI‐patients profit more than others from vaginal Er:YAG laser therapy.

## 2 Materials and Methods

### 2.1 Study Design and Population

This single‐center, single‐arm interventional study recruited women with SUI between January 2020 and May 2022 at the University Hospital Leipzig. At our institution, Er:YAG laser therapy is offered as an alternative to conservative treatment (i.e., pelvic floor muscle training, physiotherapy, incontinence pessary) to women with mild or moderate SUI. Women fulfilling the inclusion criteria were offered to participate in this study, which encompassed receiving three laser treatments with a follow‐up period of 6 months. The validated German version of the Australian Pelvic Floor Questionnaire was used to assess patient centered outcomes. All women provided written consent to participate in the study.

The inclusion criteria were: mild or moderate SUI with or without urgency symptoms, age above 18 years and the wish to receive Er:YAG‐laser therapy including follow‐up questionnaires up to 6 months after the last treatment.

The exclusion criteria were: pure urgency urinary incontinence, severe SUI (urine leakage without physical exertion), pelvic organ prolapse stage 2 or higher, pregnancy or birth in the previous 6 months, prior radical hysterectomy, exenteration or pelvic radiation therapy, neurological diseases affecting pelvic floor functions, abnormal findings in genitourinary tract (e.g., epithelial dysplasia), and previous treatment of the underlying SUI.

The study was conducted in accordance with the Declaration of Helsinki and approved by the Institutional Ethics Committee of the University Hospital of Leipzig (protocol code 547/19‐lk approved on January 28, 2020).

### 2.2 Assessment of SUI and Questionnaires

Before inclusion in this study, every woman was examined at our outpatient clinic. SUI was assessed by evaluating the degree of symptoms through a micturition diary and a clinical examination. In the latter, urine leakage was assessed by a cough test. Pad‐tests or urodynamic examinations were not routinely performed in these patients. SUI was considered mild in the case of urine leakage at pronounced increase of intraabdominal pressure (physical exertion, sneezing, coughing) and was classified as moderate in the case of urine leakage at light increase of intraabdominal pressure (walking, standing up, climbing stairs). The incontinence was classified as mixed (i.e., MUI) if additional urge symptoms were present at least once per week.

For the purpose of this analysis, the cohort was divided into two subgroups: (1) patients with pure SUI (stress incontinence without urgency) and (2) patients with MUI (stress incontinence with urgency).

Additional pelvic organ prolapse was evaluated clinically and transvaginal sonography was performed to exclude any abnormal findings in the genitourinary tract.

To quantify symptom severity, we used a validated pelvic floor questionnaire in German language (“Deutscher Beckenboden‐Fragebogen”) that quantifies symptoms of the bladder function, bowel function, sexual function, and genital prolapse [22]. This questionnaire represents the German version of the also validated Australian Pelvic Floor Questionnaire (APFQ) [23]. Women were asked to fill out the questionnaire before treatment (baseline), directly after the third treatment (posttreatment) as well as 3 and 6 months after the third treatment (3‐ and 6‐months follow‐up). At the 6‐months follow‐up visit, women were additionally asked to quantify the global improvement of their symptoms on a 0‒100 numeric rating scale.

### 2.3 Er:YAG Laser Procedure

For the laser treatment, we used a 2940 nm Er:YAG Laser System (SP Spectro, Fotona, Slovenia) with a circular full beam handpiece (R11) and angular, patterned laser beam handpiece (PS03) including the corresponding adapters and a wired laser speculum. The laser system was set in the SMOOTH mode, which is characterized by non‐ablative, thermal‐only properties (laser fluence 10 J/cm^2^, spot size 7 mm, repetition rate 1.6 Hz).

The procedure consisted of two steps. In the first step, the PS03 patterned handpiece with a 90° angular adapter was used to apply the laser beam to the anterior vaginal wall. Three passes were done at 10, 12, and 2 o'clock delivering 12 impulses every 5 mm. In the second step, the R11 full‐beam handpiece with the 360° circular adapter was used to treat the whole circumference of the vaginal wall by delivering 12 impulses every 2.5 mm in one pass.

The procedures were performed in lithotomy position and took approximately 10 min. Lidocaine vaginal gel was applied before the treatments to reduce discomfort during the procedure. The women were informed that moderate discomfort or vaginal discharge could persist for a few days, but they were not instructed to restrict themselves in any way. Every participant received a total of three treatments with a 6−8 week interval between each session [4].

### 2.4 Statistical Analysis

Statistical analyses were performed with the software environment R (version 4.4.1). Specifically, the following packages were used: “rstatix” for analysis of variance (ANOVA), “rankdifferencetest” for Kornbrot's rank difference test, “ggplot2” for graphic design.

Sample size estimation was based on the power analysis performed in a previous study which examined the minimal important difference (MID) of the APFQ [24]. According to this calculation, a total of 46 patients would be necessary to detect a MID in the questionnaire‐scores with a statistical power of 80% and an *α* of 0.05. Anticipating a loss to follow‐up rate of 20%, the minimal sample size to be included in our study was set at 58 participants.

The questionnaire‐scores at different time points were compared using Kornbrot's rank difference test, which is suitable for paired comparison of ordinal data [25]. Specifically, questionnaire results after the treatments were compared with the result scores before the treatments (i.e., baseline‐score). In a second step, patients with pure SUI and MUI were analyzed separately, as the latter has been reported to have a worse response to Er:YAG laser therapy [14].

Additionally, certain especially relevant items of the questionnaire were analyzed separately to examine the change in specific symptoms (i.e., urine leakage during exertion or intercourse, urgency, pad use, pain during intercourse, and feeling of vaginal laxity).

To explore if certain women experience more benefit than others, we compared the changes in scores (i.e., score at 6 months minus score at baseline) between different subgroups using ANOVA [26].

A *p*‐value of < 0.05 was regarded as statistically significant. Additionally, a median change in scores of approximately 1.00 was treated as the MID [24]. Consequently, the improvement was regarded to be clinically meaningful, if median scores of the cohort decreased by approximately 1.00 at 6 months after the study intervention.

## 3 Results

Between January 2020 and May 2022 a total of 61 women were enrolled in the study. Of these, 11 did not complete all three questionnaires after the study intervention and were thus excluded from the cohort (lost to follow‐up). Consequently, a total of 50 women were included into the final analysis. Their median age and BMI were 47 (IQR: 41−52) and 24.1 (IQR: 22.1−26.2), respectively. Forty‐eight (96%) women had at least one, and 30 (60%) had two or more previous vaginal births. Five (10%) had at least one previous cesarean section and two (4%) of these only had cesarean sections in their obstetric history without any vaginal birth. Symptoms of urgency were present in 17 (34%) of women, which were thus diagnosed with MUI.

**Figure 1:**
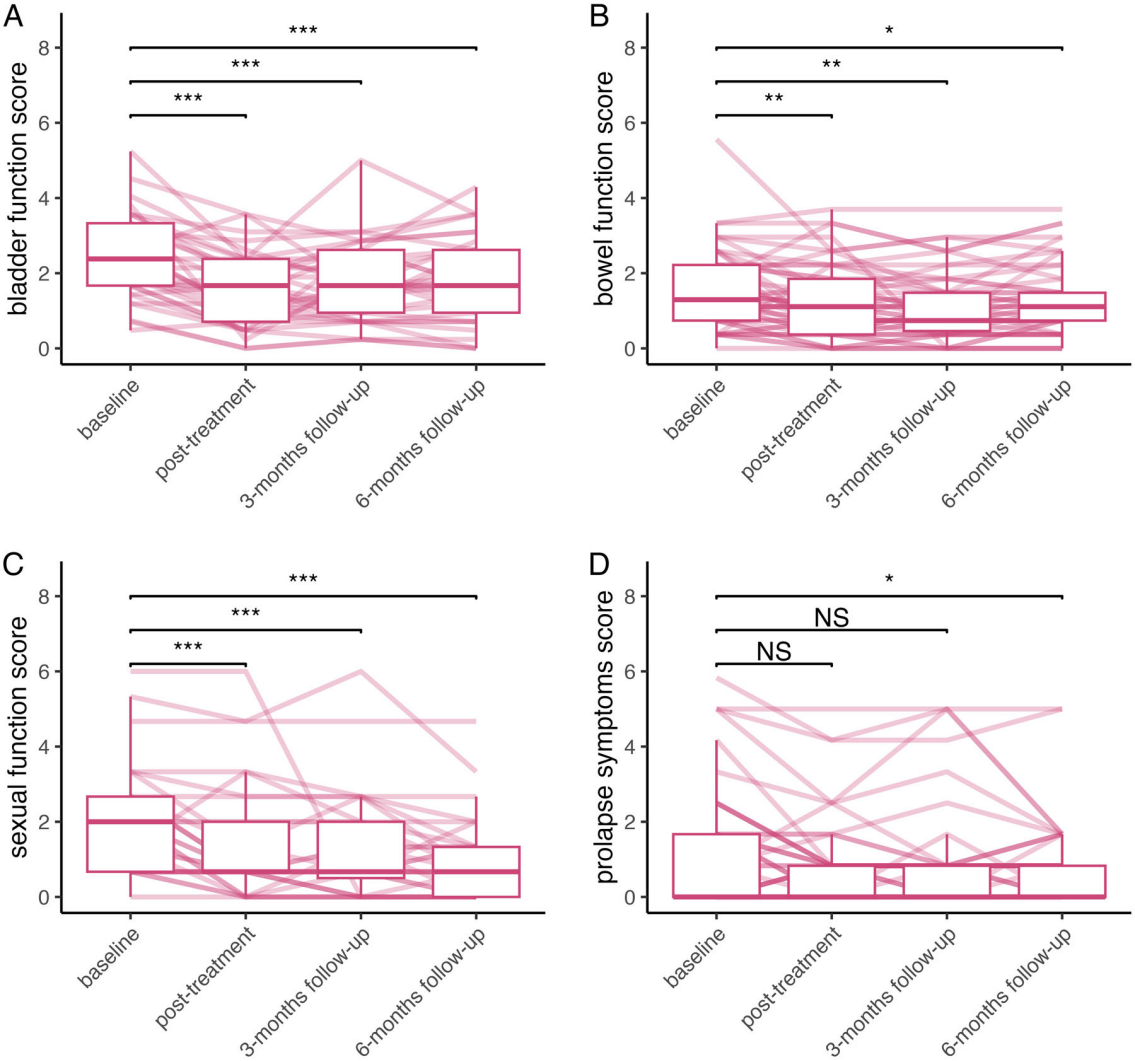
Questionnaire‐results at baseline (i.e., before laser‐therapy), posttreatment (i.e., immediately after three treatments), 3 and 6 months after treatments. The results are shown separately for (A) bladder function, (B) bowel function, (C) sexual function, and (D) prolapse symptoms. Semitransparent lines show the symptom development of every patient in the study. *p < 0.05; **p < 0.01; ***p < 0.001.

No serious adverse events leading to hospitalization or surgical intervention were observed after Er:YAG laser treatments. Every woman received all three treatments as planned and no treatment had to be interrupted due to pain or discomfort. The results of the questionnaires (both cumulative scores and specific questionnaire‐items) are shown in Tables 1 and 2 and Figure 1. At 6 months after treatment, women reported an avarage global improvement of symptoms of 49 (measured on a 0‒100 numeric rating scale). More extensively, a global improvement of ≥ 30, ≥ 50, and ≥ 70 on this scale was reported by 64%, 52%, and 34% of women, respectively.

**Table 1:** Questionnaire‐scores.

Symptom domain	Baseline	Posttreatment		3‐month follow‐up		6‐month follow‐up	
	Score	Score	*p* value	Score	*p* value	Score	*p* value
Bladder function	2.86 (1.90–3.57)	1.90 (1.19–2.62)	**< 0.001**	1.90 (1.19–2.62)	**< 0.001**	1.90 (1.19–3.33)	**< 0.001**
Bowel function	1.30 (0.74–2.22)	1.11 (0.37–1.85)	**0.006**	0.74 (0.37–1.48)	**0.002**	1.11 (0.74–1.48)	**0.048**
Sexual function	2.00 (0.67–2.67)	0.67 (0.67–2.50)	**< 0.001**	0.67 (0.00–2.00)	**< 0.001**	0.67 (0.00–1.33)	**< 0.001**
Prolapse symptoms	0.00 (0.00–1.67)	0.00 (0.00–0.83)	0.1	0.00 (0.00–0.83)	0.052	0.00 (0.00–0.83)	**0.035**

*Note:* Scores are expressed in medians and interquartile ranges. The baseline‐score represents the reference against which the scores after the treatment are tested (Kornbrot's rank difference test) and statistically significant *p*‐values are written in bold. These results are represented graphically in Figure 1. In four patients, follow‐up results for the sexual function were not available.

**Table 2:** Responses in single questions.

Question	Baseline	Posttreatment		3‐month follow‐up		6‐month follow‐up	
	*n* (%)	*n* (%)	*p* value	*n* (%)	*p* value	*n* (%)	*p* value
“Does urine leak when you rush to the toilet or can't you make it in time?” (Question 5)							
Never	10 (20%)	20 (40%)		18 (36%)		20 (40%)	
< 1/week	28 (56%)	22 (44%)	**0.0037**	27 (54%)	**0.0017**	24 (48%)	<**0.001**
≥ 1/week	12 (24%)	8 (16%)		5 (10%)		6 (12%)	
“Do you leak urine with coughing, sneezing, laughing, or exercising?” (Question 6)							
Never	5 (10%)	11 (22%)		14 (28%)		10 (20%)	
< 1/week	28 (56%)	23 (46%)		26 (52%)		31 (62%)	
≥ 1/week	12 (24%)	14 (28%)	<**0.001**	8 (16%)	<**0.001**	6 (12%)	<**0.001**
Daily	5 (10%)	2 (4%)		2 (4%)		3 (6%)	
“Do you have to wear pads because of urinary leakage?” (Question 10)							
Never	11 (22%)	16 (32%)		17 (34%)		17 (34%)	
As a precaution	11 (22%)	14 (28%)		10 (20%)		11 (22%)	
When exercising/during a cold	6 (12%)	4 (8%)	**0.006**	5 (10%)	**0.034**	8 (16%)	**0.011**
Daily	22 (44%)	16 (32%)		18 (36%)		14 (28%)	
“Does the urine leakage affect your routine activities?” (Question 14)							
No	5 (10%)	14 (28%)		13 (26%)		12 (24%)	
Slightly	20 (40%)	20 (40%)		21 (42%)		17 (34%)	
Moderately	13 (26%)	14 (28%)	<**0.001**	11 (22%)	**0.001**	13 (26%)	0.053
Greatly	12 (24%)	2 (4%)		5 (10%)		8 (16%)	
“When you get wind or flatus, does wind leak?” (Question 21)							
Never	18 (36%)	18 (36%)		19 (38%)		17 (34%)	
< 1/week	21 (42%)	26 (52%)		26 (52%)		26 (52%)	
≥ 1/week	8 (16%)	5 (10%)	0.19	4 (8%)	0.13	7 (14%)	0.34
Daily	3 (6%)	1 (2%)		1 (2%)		0 (0%)	
“Do you get an overwhelming urgency to empty your bowels?” (Question 22)							
Never	27 (54%)	37 (74%)		39 (78%)		34 (68%)	
< 1/week	19 (38%)	11 (22%)		10 (20%)		14 (28%)	
≥ 1/week	4 (8%)	2 (4%)	**0.002**	0 (0%)	**< 0.001**	2 (4%)	0.055
Daily	0 (0%)	0 (0%)		1 (2%)		0 (0%)	
“Do you leak normal stool when you don't mean to?” (Question 24)							
Never	48 (96%)	47 (94%)		47 (94%)		49 (98%)	
< 1/week	1 (2%)	3 (6%)		3 (6%)		1 (2%)	
≥ 1/week	1 (2%)	0 (0%)	0.99	0 (0%)	0.99	0 (0%)	0.16
Daily	0 (0%)	0 (0%)		0 (0%)		0 (0%)	
“Do you experience vaginal pressure/heaviness/dragging sensation?” (Question 29)							
Never	27 (54%)	30 (60%)		31 (62%)		30 (60%)	
< 1/week	13 (26%)	15 (30%)		13 (26%)		17 (34%)	
≥ 1/week	3 (6%)	1 (2%)	**0.016**	3 (6%)	**0.016**	1 (2%)	**0.022**
Daily	7 (14%)	4 (8%)		3 (6%)		2 (4%)	
“How much does your prolapse bother you?” (Question 32)							
Not at all	33 (66%)	34 (68%)		36 (72%)		37 (74%)	
Slightly	10 (20%)	10 (20%)		9 (18%)		7 (14%)	
Moderately	2 (4%)	4 (8%)	0.44	5 (10%)	0.06	4 (8%)	0.14
Greatly	5 (10%)	2 (4%)		0 (0%)		2 (4%)	
“Do you feel that your vagina is too loose or lax?” (Question 37)							
Never	23 (46%)	27 (54%)		27 (54%)		30 (60%)	
Occasionally	16 (32%)	16 (32%)	**0.024**	18 (36%)	**0.012**	15 (30%)	**0.0072**
Frequently	6 (12%)	5 (10%)		4 (10%)		4 (8%)	
Always	5 (10%)	2 (4%)		1 (2%)		1 (2%)	
“Do you experience pain with sexual intercourse?” (Question 39)							
Never	22 (44%)	32 (64%)		33 (66%)		34 (68%)	
Occasionally	24 (48%)	14 (28%)		13 (26%)		14 (28%)	
Frequently	2 (4%)	2 (4%)	**0.0016**	3 (6%)	**0.0087**	1 (2%)	**0.0028**
Always	2 (4%)	2 (4%)		1 (2%)		1 (2%)	
“Do you leak urine during sexual intercourse?” (Question 41)							
Never	33 (66%)	39 (78%)		41 (82%)		43 (86%)	
Occasionally	13 (26%)	9 (18%)		6 (12%)		5 (10%)	
Frequently	3 (6%)	0 (0%)	**0.031**	2 (4%)	**0.0027**	1 (2%)	<**0.001**
Always	1 (2%)	2 (4%)		1 (2%)		1 (2%)	

*Note:* The results at baseline represent the reference against which the results after the treatment are tested. The results are compared with tests by means of Kornbrot's rank difference test and significant *p*‐values are written in bold. The number of the questions is based on the numbering in the Australian Pelvic Floor Questionnaire.

### 3.1 Bladder Function

**Figure 2:**
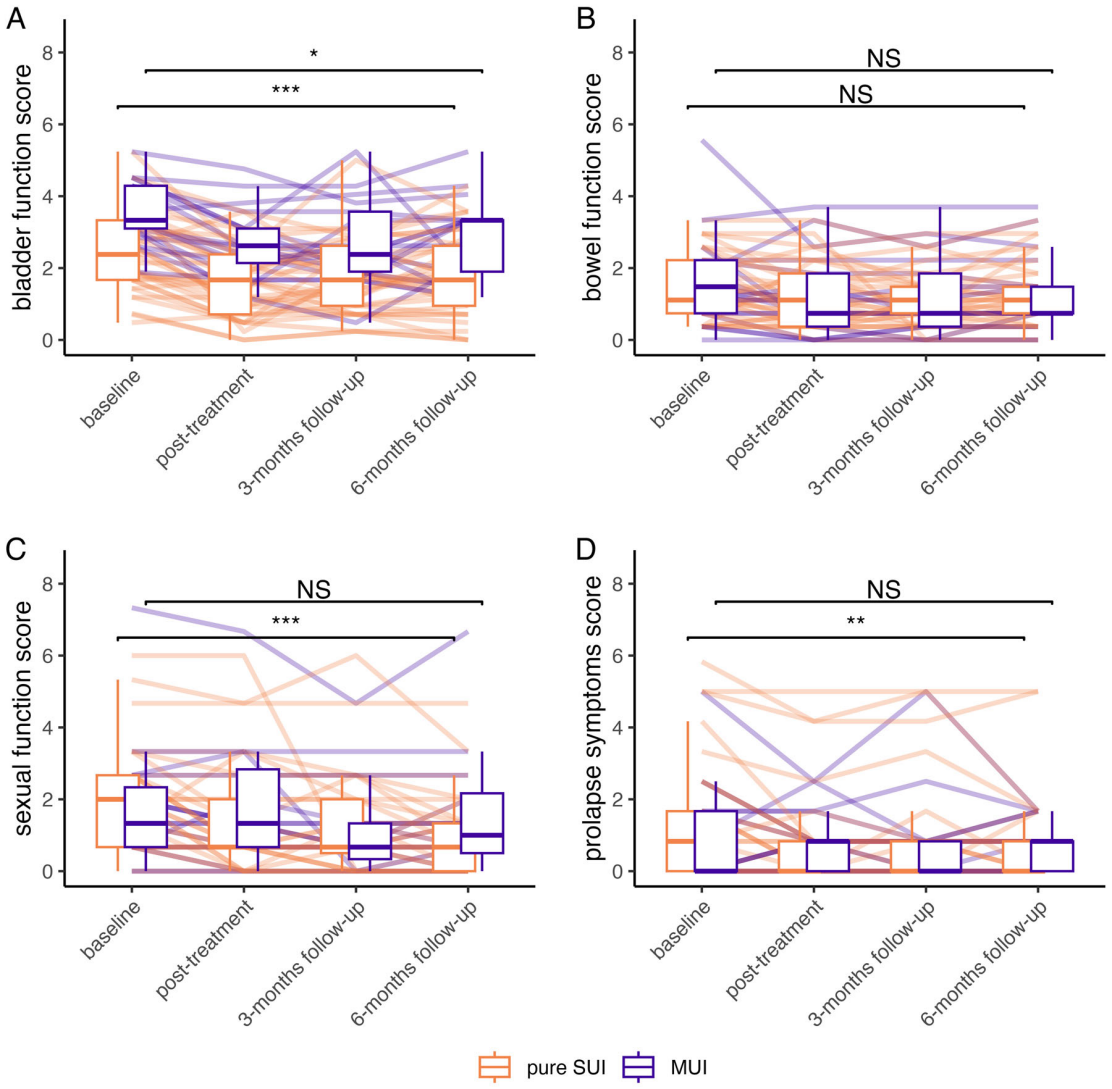
Questionnaire‐results in respect to the type of incontinence (SUI or MUI) for (A) bladder function, (B) bowel function, (C) sexual function, and (D) prolapse symptoms. Patients with MUI (box‐plots shifted to the right) show less improvement 6 months after Er:YAG laser therapy than patients with SUI (box‐plots shifted to the left). Semitransparent lines show the symptom development of every patient in the study. *p < 0.05; **p < 0.01; ***p < 0.001. MUI, mixed urinary incontinence; NS, nonsignificant; SUI, stress urinary incontinence.

Compared to baseline, bladder function scores showed statistically significant improvement up to 6 months after three Er:YAG laser treatments (median baseline‐score: 2.86; median 6‐month‐score: 1.90; *p *< 0.001; Table 1). This difference remained statistically significant in both subgroups of women with pure SUI (*p* < 0.001) and women with MUI (*p *= 0.049, Figure 2). Six months after the study intervention, bladder function scores had decreased by 0.50, 1.00, and 1.50 points in 54%, 28%, and 20% of women, respectively.

Additionally, the change in bladder function scores between baseline and 6 months after Er:YAG laser therapy were comparable among subgroups (Table 3).

**Table 3:** Comparisons of score changes between subgroups.

Symptom domain	Subgroup	Score change	Subgroup	Score change	*p* value
Bladder function	Age < 50 years	−0.48 (−0.96 to −0.23)	Age ≥ 50 years	−0.96 (−1.91 to 0.00)	0.065
	BMI < 25 kg/m^2^	−0.48 (−1.08 to 0.00)	BMI ≥ 25 kg/m^2^	−0.95 (−1.67 to −0.48)	0.34
	Vaginal births < 2	−0.73 (−1.20 to −0.12)	Vaginal births ≥ 2	−0.71 (−1.19 to −0.23)	0.95
	Pure SUI	−0.48 (−0.95 to −0.23)	MUI	−0.96 (−1.67 to 0.71)	0.54
Bowel function	**Age** < **50 years**	**0.00 (**−**0.37 to 0.37)**	**Age** ≥ **50 years**	−**0.37 (**−**1.11 to** −**0.37)**	0.034
	BMI < 25 kg/m^2^	−0.37 (−0.56 to 0.37)	BMI ≥ 25 kg/m^2^	−0.37 (−1.11 to 0.37)	0.16
	Vaginal births < 2	−0.37 (−0.74 to 0.37)	Vaginal births ≥ 2	−0.37 (−0.74 to 0.00)	0.77
	Pure SUI	−0.37 (−0.74 to 0.37)	MUI	0.00 (−0.74 to 0.00)	0.66
Sexual function	Age < 50 years	−0.67 (−1.33 to 0.00)	Age ≥ 50 years	−0.67 (−2.00 to 0.00)	0.98
	BMI < 25 kg/m^2^	−0.67 (−1.33 to 0.00)	BMI ≥ 25 kg/m^2^	−0.67 (−1.33 to 0.00)	0.44
	Vaginal births < 2	−0.67 (−2.00 to −0.66)	Vaginal births ≥ 2	−0.33 (−1.33 to 0.00)	0.096
	**Pure SUI**	−**0.67 (**−**2.00 to 0.00)**	**MUI**	**0.00 (**−**0.67 to 0.00)**	0.036
Prolapse symptoms	Age < 50 years	0.00 (−0.83 to 0.00)	Age ≥ 50 years	0.00 (−0.84 to 0.00)	0.92
	BMI < 25 kg/m^2^	0.00 (−0.83 to 0.00)	BMI ≥ 25 kg/m^2^	0.00 (−0.83 to 0.00)	0.4
	Vaginal births < 2	0.00 (−1.25 to 0.00)	Vaginal births ≥ 2	0.00 (−0.83 to 0.00)	0.57
	Pure SUI	0.00 (−0.84 to 0.00)	MUI	0.00 (0.00 to 00.00)	0.17

*Note:* Score changes (as median and IQR) represent how much the score changed over the whole study period (i.e., score at 6 months minus score at baseline) for the corresponding symptom domain and subgroup. Analysis of variance (ANOVA) was used to compare score changes across the subgroups. Statistically significant *p*‐values are represented in bold writing.

Abbreviations: IQR, interquartile range; MUI, mixed urinary incontinence; SUI, stress urinary incontinence.

At the end of the study, women reported significantly less urgency (question 5; *p *< 0.001), less urine leakage with exertion (question 6; *p *< 0.001) and utilized pads less often (question 10; *p *= 0.011). Similarly, the degree of how much incontinence affected daily routine (question 14) decreased after Er:YAG laser therapy, although only of borderline significance (*p *= 0.053) at 6 months after the study intervention (Table 2).

### 3.2 Bowel Symptoms

Bowel function scores showed a statistically significant improvement at all time points after Er:YAG laser therapy. Median scores decreased from 1.30 at baseline to 1.11 at 6 months after treatments (*p *= 0.048). This decrease was no longer statistically significant when the subgroup with pure SUI (*p *= 0.14) and the subgroup with MUI (*p *= 0.19) were analyzed separately (Figure 2).

Six months after the study intervention, bowel function scores had decreased by 0.50, 1.00, and 1.50 points in 30%, 16%, and 8% of women, respectively.

Of note, age over 50 years was associated with a stronger decrease in bowel function scores after Er:YAG laser therapy (*p *= 0.034), while no other associations between score changes and patient characteristics were observed (Table 3).

At the end of the study, women reported similar severities of flatus incontinence (question 21; *p *= 0.34), fecal incontinence (question 23; *p *= 0.16), and fecal urgency (question 22; *p *= 0.055) compared to baseline (Table 2).

### 3.3 Sexual Function

Sexual function questionnaire scores improved significantly after Er:YAG laser therapy up to 6 months after treatments (median baseline‐score: 2.00; median 6‐months‐score: 0.67; *p *< 0.001; Table 1). At 6 months after the study intervention, sexual function scores had decreased by 0.50, 1.00, and 1.50 points in 60%, 33%, and 20% of women, respectively.

In subgroup analysis, women with pure SUI showed statistically significant improvement (*p *< 0.001), while those with MUI did not (*p *= 0.21; Figure 2). Concordantly, the change in score between baseline and 6 months after treatments differed significantly between these two subgroups (*p *= 0.036). Conversely, there was no statistically significant difference among the other subgroups (Table 3).

At 6 months after Er:YAG laser therapy, women reported significantly less urine leakage (question 41; *p* < 0.001) and pain (question 39; *p* = 0.0028) during intercourse. Additionally, the feeling of the vagina being too loose or lax occurred significantly less often (question 37; *p* = 0.0072).

### 3.4 Prolapse Symptoms

Median scores in prolapse symptoms were lower than scores in other symptom complexes (Table 1). At baseline, 23 (46%) women reported some symptoms due to genitourinary prolapse, while 27 (54%) were asymptomatic (i.e., prolapse symptoms score at 0.00). At 6 months after Er:YAG laser therapy, scores of prolapse symptoms were significantly lower compared to scores at baseline (*p* = 0.034). These scores decreased by 0.50, 1.00, and 1.50 points in 30%, 18%, and 18% of women, respectively.

This decrease was significant in the subgroup of women with pure SUI (*p *= 0.0092) but not in the women with MUI (*p *= 0.8). Of note, scores at posttreatment (*p *= 0.1) and at 3 months (*p *= 0.052) were not significantly different from baseline (Table 1). The change in scores between baseline and 6 months after treatment were comparable among subgroups (Table 3).

At the end of the study, we observed a statistically significant reduction of vaginal pressure/heaviness (question 29; *p *= 0.022). However, women reported similar overall bothersomeness caused by prolapse (question 32; *p *= 0.14) compared to before vaginal Er:YAG laser therapy (Table 2).

## 4 Discussion

This study observed that women with SUI undergoing three treatment sessions with vaginal Er:YAG laser experienced an improvement in symptoms linked to bladder function and sexual function. To a lesser degree, bowel function and prolapse symptoms were ameliorated as well. Consequently, these results support the use of this laser‐based therapy in patients with SUI and are in line with the findings in previous studies [14, 15, 16, 18, 27, 28].

Er:YAG laser in the appropriate settings has been shown to increase temperature in the vaginal mucosa without leading to tissue destruction [27]. This non‐ablative laser application leads to a temperature build‐up reaching, but not exceeding, 65°C. This specific range causes a shortening of collagen fibers without denaturation [29] and promotes neocollagenesis [10, 12, 14]. Consequently, it has been suggested that vaginal Er:YAG laser therapy could improve SUI, which is thought to be caused by changes in collagen ultrastructure in the pelvic floor [30] and hypermobility of both bladder and urethra [31]. In fact, several studies already showed the effect of vaginal Er:YAG laser therapy on both bladder neck mobility [16] and vaginal cross sectional area [17].

Previous studies focused on incontinence symptoms quantified by means of standardized questionnaires (e.g., ICIQ‐UI/SF, UDI‐6, IID‐7, KHQ‐UI) and all reported statistically significant improvements after Er:YAG laser therapy [13, 14, 16, 27]. Interestingly, questionnaire results in these studies suggested that symptoms related to sexual dysfunction [17, 18, 19] or pelvic organ prolapse [16, 18] improve as well. Importantly, these results have been confirmed in randomized controlled trials comparing Er:YAG laser therapy with either sham laser therapy [18, 19] or physical therapy [28].

Yet it must be kept in mind that the reported improvements of questionnaire‐scores may not necessarily reflect a meaningful improvement of the women's symptoms. This is due to the fact that cumulative scores only give a rough approximation of the true measures (e.g., discomfort caused by SUI) [20]. Additionally, statistical significance does not automatically translate into a proof of benefit. Discussing changes in questionnaire scores by means of the MID, instead of exclusively by statistical significance, would have improved the validity of the conclusion on whether Er:YAG laser therapy benefits women with SUI [32]. To the best of our knowledge, only one previous study (additionally to the present one) interpreted the effects of vaginal Er:YAG laser therapy by means of an appropriate MID. This prospective cohort study observed, setting the MID at a 30% decrease of the ICIQ‐UI‐score, a significant and meaningful improvement [33].

Our study observed a statistically significant improvement of all symptom complexes related to the pelvic floor (bladder function, bowel function, sexual function, and prolapse symptoms) after three vaginal Er:YAG laser treatments. However, the changes in bowel function sores and prolapse symptom scores were below the MID for this questionnaire [24] and thus likely not reflecting a meaningful improvement. However, it should be kept in mind that scores for bowel function and prolapse symptoms were relatively low at baseline, thus limiting the maximum possible decrease in score points due to a floor effect [20]. Consequently, vaginal Er:YAG laser therapy may be mostly effective in improving bladder and sexual symptoms in women with SUI, as the improvements in these symptom domains were both statistically significant and clinically meaningful (Tables 1 and 2).

Overall, the improvement in questionnaire scores was weaker or even absent in patients with MUI (i.e., SUI with urge symptoms), which has been shown in a previous study [14]. Especially symptoms related to sexual function improved significantly less when patients in this study had MUI. Furthermore, women's age, overweight, and previous vaginal births did not seem to influence the effect of Er:YAG laser therapy on pelvic floor symptoms in this cohort. This finding stands in contrast to those of another cohort study, which did in fact observe that age and BMI influence the effect of vaginal Er:YAG laser therapy on SUI‐symptoms [33]. Only a statistically significant association between women's age and the change in bowel function scores could be observed (Table 1), even though likely not meaningful as markedly below the MID [24].

Our study has some important limitations. First, the lack of a control group receiving a placebo treatment limits the conclusions that can be drawn from our results. Second, there was a relevant number of women lost to follow‐up. This could have led to a bias skewing the results and ultimately favoring the study intervention. Third, the number of patients was small, thus limiting the conclusions of nonsignificant relationships. Specifically, the less significant and nonsignificant results in the MUI‐subgroup, which included only 17 cases, may have been caused by the small size limiting statistical power. Moreover, the size of the cohort was too small to test for multiple interactions between women's characteristics and the success of the study intervention (i.e., multivariable regression). Lastly, the primary outcomes in this study are based solely on questionnaire results (i.e., questionnaire scores) and no objective measurements (e.g., urodynamic testing) were performed. Consequently, conclusions drawn by this study can only be applied on the patient‐reported burden of SUI.

Nevertheless, the size of this cohort was comparable to those of other studies in this field of research [14, 16, 27] and, importantly, was powered to detect a MID for the questionnaire used to measure the outcome. Additionally, we also examined the development of specific symptoms by analyzing the questionnaire item, thus making these results more informative than mere cumulative questionnaire scores. In summary, these results may add new insights into the effects of vaginal Er:YAG laser therapy in women with SUI.

## 5 Conclusions

In this study, we showed that women with SUI receiving vaginal Er:YAG laser therapy experienced a statistically significant and meaningful improvement of pelvic floor symptoms associated with bladder and sexual function. However, the potentially beneficial effect of this treatment may be limited by additional urgency symptoms (i.e., MUI), especially in symptoms of sexual function.

## Author Contributions

**Pavel Pilát and Bahriye Aktas:** conceptualization. **Pavel Pilát and Bahriye Aktas:** methodology. **Massimiliano Lia:** software. **Pavel Pilát and Bahriye Aktas:** validation. **Massimiliano Lia:** formal analysis. **Pavel Pilát:** investigation. **Tom Kempe and Bahriye Aktas:** resources. **Pavel Pilát:** data curation. **Massimiliano Lia:** writing–original draft preparation. **Bahriye Aktas, Laura Weydandt, Kaven Baessler, Tom Kempe, and Pavel Pilát:** writing–review and editing. **Massimiliano Lia:** visualization. **Bahriye Aktas:** supervision. **Bahriye Aktas:** project administration. All authors have read and agreed to the published version of the manuscript.

## Conflicts of Interest

The authors declare no conflicts of interest.

## References

[1] K. M. Luber , “The Definition, Prevalence, and Risk Factors for Stress Urinary Incontinence,” supplement, Reviews in Urology 6, no. S3 (2004): 3–9. PMC1472862 16985863

[2] B. T. Haylen , D. de Ridder , R. M. Freeman , et al., “An International Urogynecological Association (IUGA)/International Continence Society (ICS) Joint Report on the Terminology for Female Pelvic Floor Dysfunction,” International Urogynecology Journal 21, no. 1 (2010): 5–26, 10.1007/s00192-009-0976-9. 19937315

[3] F. Shabani , M. Montazeri , A. Alizadeh , et al., “The Relationship Between Urinary Incontinence With Sexual Function and Quality of Life in Postmenopausal Women,” Post Reproductive Health 29, no. 1 (2023): 15–23, 10.1177/20533691231155734. 36749321

[4] G. Naumann , T. Aigmüller , W. Bader , et al., “Diagnosis and Therapy of Female Urinary Incontinence. Guideline of the DGGG, OEGGG and SGGG (S2k‐Level, AWMF Registry No. 015/091, January 2022): Part 1 With Recommendations on Diagnostics and Conservative and Medical Treatment,” Geburtshilfe und Frauenheilkunde 83, no. 4 (2023): 377–409, 10.1055/a-1967-1726. 37034417 PMC10076094

[5] J. M. Holroyd‐Leduc and S. E. Straus , “Management of Urinary Incontinence in Women: Scientific Review,” Journal of the American Medical Association 291, no. 8 (2004): 986–995, 10.1001/jama.291.8.986. 14982915

[6] E. S. Rovner and A. J. Wein , “Treatment Options for Stress Urinary Incontinence,” supplement, Reviews in Urology 6, no. S3 (2004): S29–S47. PMC1472859 16985862

[7] S. Cavkaytar , M. K. Kokanali , H. O. Topcu , O. S. Aksakal , and M. Doğanay , “Effect of Home‐Based Kegel Exercises on Quality of Life in Women With Stress and Mixed Urinary Incontinence,” Journal of Obstetrics and Gynaecology 35, no. 4 (2015): 407–410, 10.3109/01443615.2014.960831. 25264854

[8] B. D. O'Leary , A. McCreery , A. E. Redmond , and D. P. Keane , “The Efficacy and Complications of Retropubic Tension‐Free Vaginal Tapes After 20 Years: A Prospective Observational Study,” BJOG: An International Journal of Obstetrics & Gynaecology 130, no. 1 (2023): 107–113, 10.1111/1471-0528.17282. PMC10087949 36053874

[9] U. Ulmsten , C. Falconer , P. Johnson , et al., “A Multicenter Study of Tension‐Free Vaginal Tape (TVT) for Surgical Treatment of Stress Urinary Incontinence,” International Urogynecology Journal and Pelvic Floor Dysfunction 9, no. 4 (1998): 210–213, 10.1007/BF01901606. 9795826

[10] B. Drnovšek‐Olup , M. Beltram , and J. Pižem , “Repetitive Er:YAG Laser Irradiation of Human Skin: A Histological Evaluation,” Lasers in Surgery and Medicine 35, no. 2 (2004): 146–151, 10.1002/lsm.20080. 15334619

[11] B. M. Hantash , A. A. Ubeid , H. Chang , R. Kafi , and B. Renton , “Bipolar Fractional Radiofrequency Treatment Induces Neoelastogenesis and Neocollagenesis,” Lasers in Surgery and Medicine 41, no. 1 (2009): 1–9, 10.1002/lsm.20731. 19143021

[12] L. Hympanova , K. Mackova , M. El‐Domyati , et al., “Effects of Non‐Ablative Er:YAG Laser on the Skin and the Vaginal Wall: Systematic Review of the Clinical and Experimental Literature,” International Urogynecology Journal 31, no. 12 (2020): 2473–2484, 10.1007/s00192-020-04452-9. 32780174

[13] N. Fistonić , I. Fistonić , A. Lukanovič , Š. F. Guštek , I. S. B. Turina , and D. Franić , “First Assessment of Short‐Term Efficacy of Er:YAG Laser Treatment on Stress Urinary Incontinence in Women: Prospective Cohort Study,” supplement, Climacteric 18, no. S1 (2015): 37–42, 10.3109/13697137.2015.1071126. 26366799

[14] C. T. Erel , D. Inan , and A. Mut , “Predictive Factors for the Efficacy of Er:YAG Laser Treatment of Urinary Incontinence,” Maturitas 132 (2020): 1–6, 10.1016/j.maturitas.2019.11.003. 31883657

[15] U. B. Ogrinc , S. Senčar , and H. Lenasi , “Novel Minimally Invasive Laser Treatment of Urinary Incontinence in Women,” Lasers in Surgery and Medicine 47, no. 9 (2015): 689–697, 10.1155/2013/567375. 26388213 PMC5396289

[16] K.‐L. Lin , S.‐H. Chou , and C.‐Y. Long , “Effect of Er:YAG Laser for Women With Stress Urinary Incontinence,” BioMed Research International 2019 (2019): 7915813, 10.1155/2019/7915813. 30766886 PMC6350556

[17] C.‐Y. Long , P.‐C. Wu , H.‐S. Chen , et al., “Changes in Sexual Function and Vaginal Topography Using Transperineal Ultrasound After Vaginal Laser Treatment for Women With Stress Urinary Incontinence,” Scientific Reports 12, no. 1 (2022): 3435, 10.1038/s41598-022-06601-0. 35236871 PMC8891315

[18] M. Blaganje , D. Šćepanović , L. Žgur , I. Verdenik , F. Pajk , and A. Lukanović , “Non‐Ablative Er:YAG Laser Therapy Effect on Stress Urinary Incontinence Related to Quality of Life and Sexual Function: A Randomized Controlled Trial,” European Journal of Obstetrics & Gynecology and Reproductive Biology 224 (2018): 153–158, 10.1016/j.ejogrb.2018.03.038. 29604548

[19] B. A. O'Reilly , V. Viereck , C. Phillips , et al., “Vaginal Erbium Laser Treatment for Stress Urinary Incontinence: A Multicenter Randomized Sham‐Controlled Clinical Trial,” International Journal of Gynecology & Obstetrics 164, no. 3 (2024): 1184–1194, 10.1002/ijgo.15222. 37927157

[20] L. Tesio , S. Scarano , S. Hassan , D. Kumbhare , and A. Caronni , “Why Questionnaire Scores Are Not Measures: A Question‐Raising Article,” American Journal of Physical Medicine & Rehabilitation 102, no. 1 (2023): 75–82, 10.1097/PHM.0000000000002028. 35700126 PMC9770109

[21] L. Frank , E. Basch , and J. V. Selby , “The PCORI Perspective on Patient‐Centered Outcomes Research,” Journal of the American Medical Association 312, no. 15 (2014): 1513–1514, 10.1001/jama.2014.11100. 25167382

[22] K. Baessler and C. Kempkensteffen , “Validierung Eines Umfassenden Beckenboden‐Fragebogens Für Klinik, Praxis Und Forschung,” Gynäkologisch‐geburtshilfliche Rundschau 49, no. 4 (2009): 299–307, 10.1159/000301098. 20530945

[23] K. Baessler , S. M. O'Neill , C. F. Maher , and D. Battistutta , “A Validated Self‐Administered Female Pelvic Floor Questionnaire,” International Urogynecology Journal 21, no. 2 (2010): 163–172, 10.1007/s00192-009-0997-4. 19756341

[24] K. Baessler , A. Mowat , C. F. Maher , “The Minimal Important Difference of The Australian Pelvic Floor Questionnaire,” International Urogynecology Journal 30 (2019): 115–122, 10.1007/s00192-018-3724-1. 30088031

[25] D. E. Kornbrot , “The Rank Difference Test: A New and Meaningful Alternative to the Wilcoxon Signed Ranks Test for Ordinal Data,” British Journal of Mathematical and Statistical Psychology 43, no. 2 (1990): 241–264, 10.1111/j.2044-8317.1990.tb00939.x.

[26] J. Jamieson , “Analysis of Covariance (ANCOVA) With Difference Scores,” International Journal of Psychophysiology 52, no. 3 (2004): 277–283, 10.1016/j.ijpsycho.2003.12.009. 15094250

[27] N. Fistonić , I. Fistonić , Š. F. Guštek , et al., “Minimally Invasive, Non‐Ablative Er:YAG Laser Treatment of Stress Urinary Incontinence in Women: A Pilot Study,” Lasers in Medical Science 31, no. 4 (2016): 635–643, 10.1007/s10103-016-1884-0. 26861984 PMC4851697

[28] L. C. da Fonseca , F. B. A. Giarreta , T. V. Peterson , et al., “A Randomized Trial Comparing Vaginal Laser Therapy and Pelvic Floor Physical Therapy for Treating Women With Stress Urinary Incontinence,” Neurourology and Urodynamics 42, no. 7 (2023): 1445–1454, 10.1002/nau.25244. 37449372

[29] V. T. Wood , C. E. Pinfildi , M. A. I. Neves , N. A. Parizoto , B. Hochman , and L. M. Ferreira , “Collagen Changes and Realignment Induced by Low‐Level Laser Therapy and Low‐Intensity Ultrasound in the Calcaneal Tendon,” Lasers in Surgery and Medicine 42, no. 6 (2010): 559–565, 10.1002/lsm.20932. 20662033

[30] L. Han , L. Wang , Q. Wang , H. Li , and H. Zang , “Association Between Pelvic Organ Prolapse and Stress Urinary Incontinence With Collagen,” Experimental and Therapeutic Medicine 7, no. 5 (2014): 1337–1341, 10.3892/etm.2014.1563. 24940435 PMC3991483

[31] Y. Chen , L. Peng , M. Liu , H. Shen , and D. Luo , “Diagnostic Value of Transperineal Ultrasound in Patients With Stress Urinary Incontinence (SUI): A Systematic Review and Meta‐Analysis,” World Journal of Urology 41 (2023): 687–693, 10.1007/s00345-022-04264-0. 36598556

[32] R. Jaeschke , J. Singer , and G. H. Guyatt , “Measurement of Health Status,” Controlled Clinical Trials 10, no. 4 (1989): 407–415, 10.1016/0197-2456(89)90005-6. 2691207

[33] I. Fistonić and N. Fistonić , “Baseline ICIQ‐UI Score, Body Mass Index, Age, Average Birth Weight, and Perineometry Duration as Promising Predictors of the Short‐Term Efficacy of Er:YAG Laser Treatment in Stress Urinary Incontinent Women: A Prospective Cohort Study,” Lasers in Surgery and Medicine 50 (2018): 636–643, 10.1002/lsm.22789. 29360142

